# The Electric Honeycomb; an investigation of the Rose window instability

**DOI:** 10.1098/rsos.170503

**Published:** 2017-10-04

**Authors:** Muhammad Shaheer Niazi

**Affiliations:** Lahore College of Arts and Sciences, Lahore, Punjab, Pakistan

**Keywords:** instability, electrohydrodynamics, Rose window

## Abstract

The Rose window instability is a little-explored electrohydrodynamic instability that manifests when a layer of low-conducting oil is placed in an electric field generated by corona discharge in a point-to-plane configuration. Above a critical voltage, the instability starts as a single dimple in the oil layer right below the point electrode and subsequently evolves into a characteristic pattern of polygonal cells. In this study, we experimentally explore governing parameters that guide the instability and document geometric attributes of the characteristic cellular pattern. The driving force for the instability has been attributed to the buildup of charged ions which in turn apply an electric pressure on the oil surface. We confirm the charged surface distribution using thermal imaging and demonstrate that the instability can be locally inhibited by preventing charge buildup under an ion shadow.

## 1. Introduction

**Figure 1.**
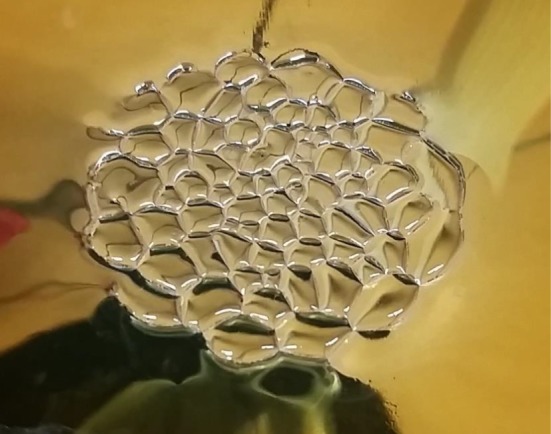
Close up of the Rose-window instability (RWI) as observed in our experiments.

Controlled spreading of droplets and liquid films over a solid substrate has widespread use in applications such as coating, printing, micro- and bio-fluidics to name a few. Recently, it has been proposed that corona discharge can be an effective technique for controlled droplet spreading [1,2]. Corona discharge is effected by the ionization of air in the vicinity of a sharp-tipped conductor carrying a high voltage. Free charges created in the process accumulate atop the droplet that rests on a conducting plate and subsequently help guide the motion of the droplet under an applied electric field. However, as the droplet spreads to a film, the air–liquid interface can be rendered unstable and under certain conditions spontaneously evolves into a stable pattern of polygonal cells, each of a size several times larger than the thickness of the film (figure 1). The instability was nicknamed the Rose window instability (RWI) by Pérez [3], who first identified that it occurs only in low-conducting liquids. Subsequently, the RWI has been explored in more detail both experimentally and theoretically by only a handful of researchers. Vega & Pérez [4] used linear stability analysis to obtain a theoretical estimate of the critical voltage at which RWI initiates for an air–ohmic liquid interface subject to a perpendicular electric field. The results were verified against experiments in a subsequent study [5], wherein the RWI was investigated experimentally for low-conducting fluids in both the ohmic and non-ohmic regimes. More recent theoretical investigations have extended the stability analysis to consider the role of liquid motion [6] and the presence of an insulating liquid [7]. In this study, we extend experimental investigation of the RWI and explore the parametric space wherein the instability manifests itself. We also document geometric attributes of the instability pattern and finally prove the role of charged ions as the key ingredient that drives the instability.

## 2. Material and methods

**Figure 2.**
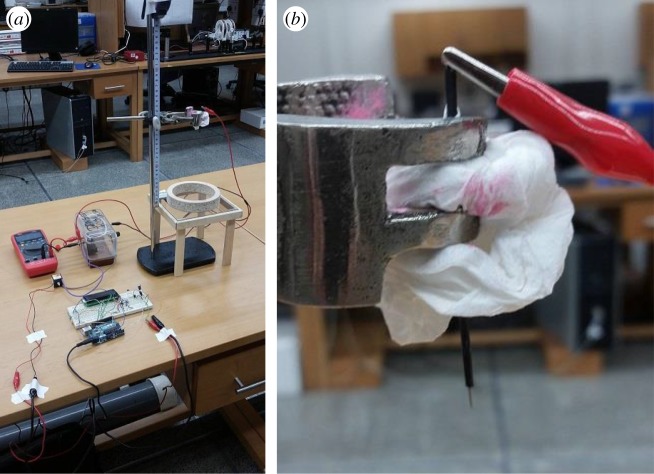
Experimental set-up. (*a*) A glass plate coated with FTO serves as the plate electrode while a cylindrical barrier prevents oil leakage. (*b*) Needle body insulated with heat sink tubing, to minimize stray fields and corona leakage.

Early research into the RWI [3] identified the key conditions needed for the instability to reveal itself: a fluid with very low conductivity which makes it a dielectric barrier to the charge that aspires to flow to the underlying conducting plate; a point electrode to have a high curvature, like a needle point, to create an injection electrode that can ionize air molecules; and a pulsed DC high voltage supply (EMCO Q101-5). The experimental set-up for this study consists of a custom-made wood table to support the plate electrode (figure 2*a*). The injection electrode is a sandpapered steel needle insulated on the body to keep away stray fields and corona leakage (figure 2*b*). The needle is hoisted on a burette stand and a vernier scale used to measure adjusted pin heights. The plate electrode consists of an FTO (fluorinated tin oxide) coated glass electrode [4] which aids in imaging. A toroidal wooden collar pasted on the glass electrode helps confine the low-conducting fluid, which for our study is machine oil (approx. 890 kg m^−3^). The high voltage supply used for the experiments provides variable voltage in the range of 0–10 kV DC at 75–500 kHz. Voltage and current were measured using UNI-T UT60 and an EMCO V1G HV divider (1000 : 1). Schlieren photography using shadowgraphy was also done using a 5.1 inch parabolic mirror.

## 3. Results

### 3.1. Critical voltage

**Figure 3.**
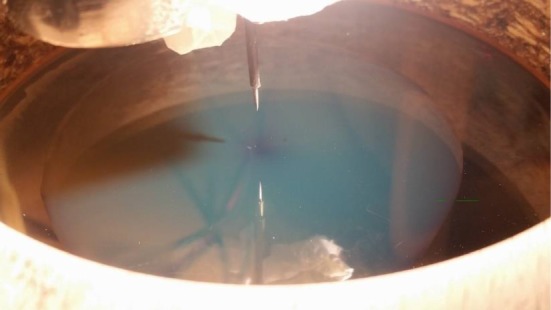
A surface dimple which marks the initiation of the RWI. Wooden collar is also visible.

We investigate the critical voltage at which the RWI manifests itself as a function of the pin height and liquid layer thickness. Here, we define the start of the instability as the formation of a single dimple in the oil layer (figure 3) which is readily detected by placing a piece of paper under the glass electrode. Owing to the refractive properties of the dimple, a shadow is formed on the paper signifying its presence.

**Figure 4.**
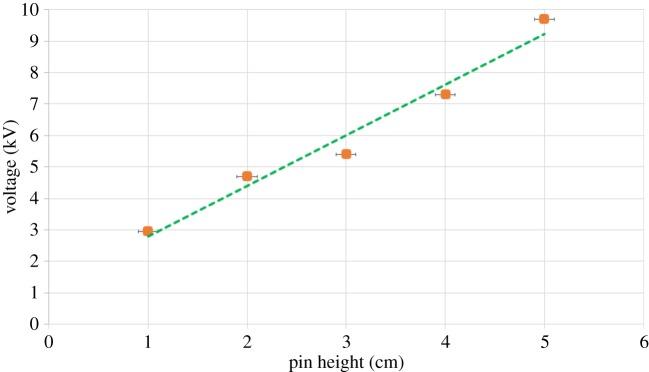
Variation of critical voltage with pin height. The dashed line shows a linear-fit to the experimental data. Horizontal error bars in accordance to error factor of the vernier caliper. Vertical error bars too small to be seen.

**Figure 5.**
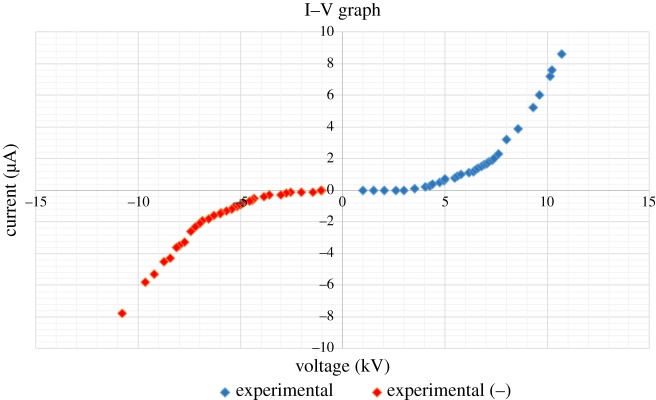
Current–voltage characteristic for the RWI instability. Note that no current is recorded before the threshold voltage is reached which marks the initiation of the instability/corona. Pin height 1 cm, layer thickness roughly 2 mm.

To investigate the role of pin height, we start with an oil layer of thickness approximately 1 mm which is subsequently held constant. Next, we increment the pin height from 1 to 5 cm (using a step of 1 cm) and measure the voltage at which the instability initiates. We find that the critical voltage increases linearly with an increase in pin height (figure 4). This trend is in accord with experimental results reported by Pérez [3], who attributed the instability to the electric pressure acting at the air–liquid interface. With increasing pin height *L*, the voltage *V* needed to obtain the same strength of electric field *E* (and correspondingly electric pressure *P* ∼ *E*2) must increase linearly in accord with *E* ∼ *V*/*L* as we can assume the electric field at the oil surface to be homogeneous due to a small ratio of the pin height and instability radius. Using an I–V graph like in figure 5, one can also determine critical voltage versus pin height.

**Figure 6.**
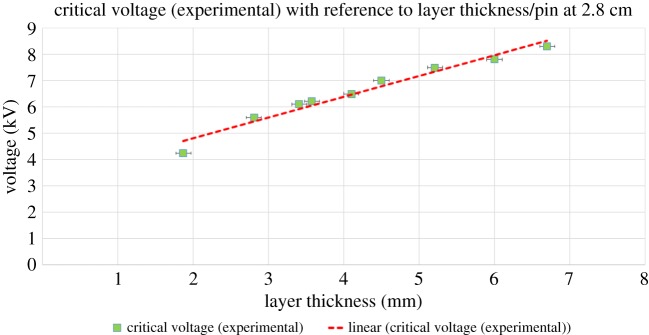
Variation of critical voltage with liquid layer thickness. The dashed line shows a linear-fit to the experimental data. Horizontal error bars in accordance to error factor of the vernier caliper. Vertical error bars too small to be seen. Pin height 2.8 cm.

**Figure 7.**
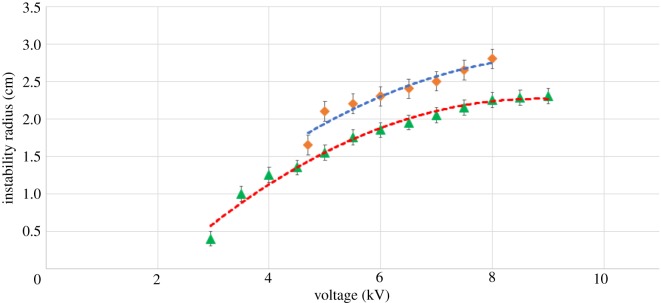
Instability radius versus injection voltage. The triangles represent data for a pin height of 1 cm and the diamonds represent data for a pin height of 2 cm. Dashed lines are best-fit curves.

Next, we investigate the role of the liquid film thickness *d* (figure 6). Previous studies have explored the variation of critical voltage with *d* within a narrow range of values from *d* ∼ 0.6 to 1.6 mm. Here, we explore a much larger thickness range from *d* ∼ 1.9 to 6.8 mm. The thickness was gradually increased by adding a certain amount of oil and measuring the depth using a vernier caliper. The pin height was kept constant at 2.8 cm. We find that even for the extended range of values the critical voltage *V* increases roughly linearly with *d* in keeping with incomplete results in fig. 7 of Pérez [3], where the critical voltage increases from approximately 2 mm to approximately 2.5 mm with ‘point positive’. So we note that after a thickness of approximately 2 mm, the critical voltage starts increasing as shown by our results and two sets of values in Pérez [3].

### 3.2. Geometric characteristics of the Rose window instability

**Figure 8.**
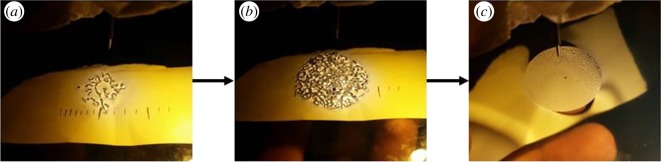
Evolution of the RWI with increasing voltage beyond the critical threshold. These images were obtained with a pin height of 1 cm and the corresponding threshold voltage is 2.95 kV. The paper placed below the system is a means to view the instability better. The lines are merely marks to record size. (*a*) RWI at around 3.2 kV. (*b*) With increasing voltage (approx. 5 kV), the instability develops with a defined, yet irregular, boundary. (*c*) At 10 kV, the cells reduce to tiny polygons within a more well defined circular instability radius.

We explore geometric characteristics of the RWI for our point–plane configuration which, to our knowledge, has not yet been explored in detail. We observe that if the voltage is held constant, beyond the critical voltage, the unstable air–liquid interface settles into a stable configuration of polygonal cells with a near circular boundary. We explore the spatial extent of the cell pattern, measured as the radial distance from the pin to the edge of the cell pattern, which we refer to as the instability radius (figures 7 and 8). We measure the instability radius as a function of the applied voltage for two different pin heights. For each case, the instability radius increases rapidly at first, but soon saturates to a near constant value (figure 7). We attribute this response to the fact that the electric field lines emanating from the injecting electrode spread at a certain deviation angle from each other, which in turn leads to an inhomogeneity of the electric field that acts on the surface of the oil. As we increase the voltage, the field density increases which acts to reduce the inhomogeneity of the electric field as neighbouring lines deviate less from each other.

**Figure 9.**
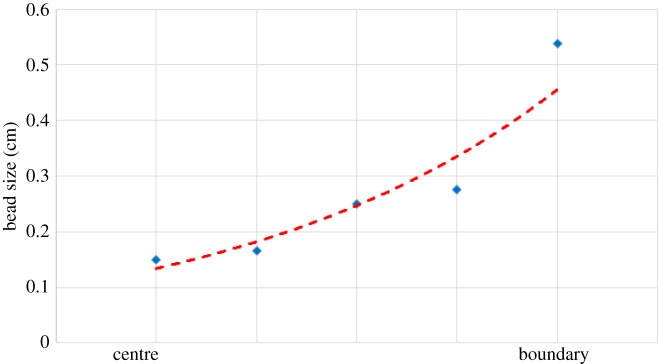
Radial variation of cell size. The radial distance is measured from the tip of the injecting electrode. Pin height 3 cm at 10 kV.

Radial heterogeneity in the electric field strength also manifests in the size of the polygonal cells that form due to RWI. In the tip–plane configuration, the electric field is strongest right underneath the tip and reduces radially away from the tip. Correspondingly, we find that the cell sizes vary radially with the smallest cells below the tip and larger cells at the boundary (figure 9). Cell sizes would be related to field density owing to the gradient of electrostatic pressure on the oil due to the ionic bombardment.

### 3.3. Surface charge distribution

**Figure 10.**
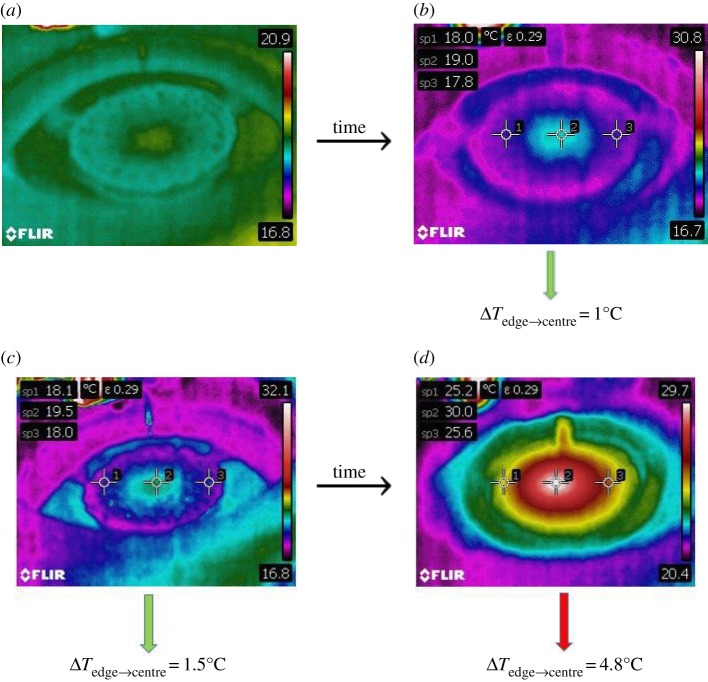
Thermal imaging of the RWI. (*a*) Start of instability, almost uniform temperature gradient (18.6°C). (*b*) Half a minute after initiation. (*c*) Image obtained about a minute after initiation of the instability. (*d*) Image obtained about 5 min after initiation of the instability. For each case, point 2 marks the centre of the instability which occurs right underneath the tip of the injecting electrode. For both (*c*) and (*d*), a radial temperature gradient is evident with temperatures rising as we traverse from the centre to the boundary of the instability, roughly marked by points 2 and 3.

**Figure 11.**
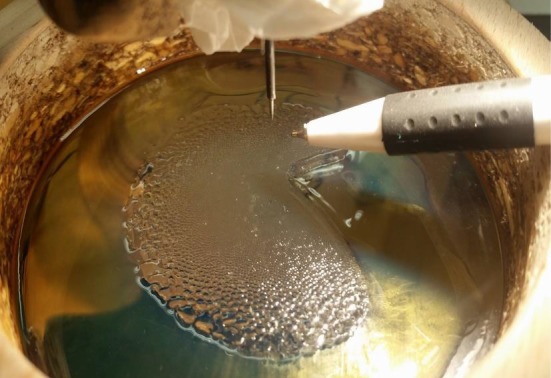
Local inhibition of the RWI by creation of an ion shadow that prevents surface charge built-up around the insulating pen. The curvature of the shadow results from the shape of the fountain-shaped electric field in the tip–plane configuration.

**Figure 12.**
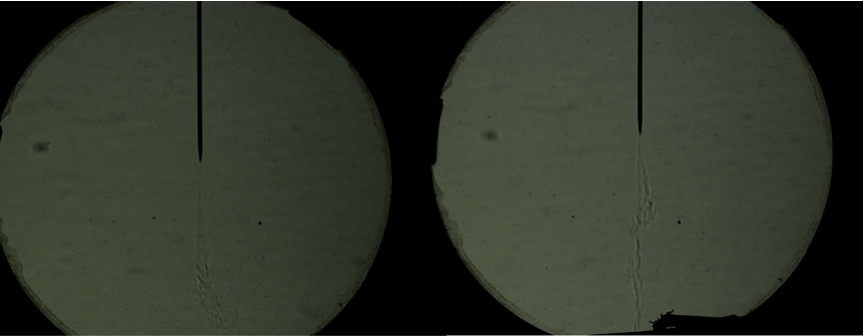
Schlieren images of the corona streamer emanating from the point electrode (needle). Using schlieren photography we can visualize variations in density/temperature in fluid mediums such as air in our case.

The driving force for the RWI has been attributed to the action of the electric field on the surface charges that accumulate atop the low-conducting oil layer. As the charged ions bombard the surface, they carry a certain energy, part of which must be deposited onto the surface of the oil and over time should lead to a significant shift in the oil temperature. Thus, to reveal the distribution of these surface charges, we resort to thermal imaging. Using a thermal camera, we take images of the oil surface at four instances: one at the start, then at half a minute and a minute after the initiation of the instability and another after about 5 min (figure 10). The radial temperature gradient evident in these images serves to verify the assumption of a radially inhomogeneous charge distribution, which in turn manifests radial variations in the geometric attributes of the polygonal cells as discussed earlier. To further strengthen the role of surface charges as the key ingredient responsible for the RWI, we shield part of the oil surface from charge accumulation by creating an ion shadow. An ion shadow forms when an object blocks the path of the charged ions towards the surface of the oil. Even thin objects, like paper, are sufficient to produce a sharp shadow that inhibits charge accumulation on the oil surface. In the absence of surface charges the instability is locally inhibited, as evident from figure 11. Even metal objects produce the same effect when placed in the path of the charged ions. Using Schlieren photography (figure 12), we imaged the corona streamer which shows that the streamer and gas flow stay roughly the same size as the needle diameter, while the instability continues to occur at a larger radius.

### 3.4. Current–voltage characteristic

Lastly, we investigate the evolution of the I–V curve in our experimental set-up. We note that the polygonal cells of the RWI form to allow for the transfer of the charged ions atop the oil to the grounded plate electrode. Thus, one might visualize the instability as a breakdown of the dielectric barrier of oil which prevents electrical conduction between the injecting electrode and the grounded plate. Our measured I–V curve confirms this notion. We find that before the instability initiates, we measure zero current at the grounded electrode. As the instability forms, ions are able to migrate to the grounded electrode and we correspondingly measure an increase in current. With increasing voltage, the depressions created by the RWI grow rapidly, allowing more ions to ground and correspondingly we record an exponential increase of the I–V curve.

## 4. Discussion

If we compare the RWI to other instabilities, we see that they have something in common, that is to achieve an equilibrium in the system. The RWI achieves a flow of current out of the system by essentially completing the circuit. It does that by creating the polygonal patterns. If we look at the case in figure 11 and instead place a wire connected to an ammeter in the flow of ions, then we notice an inflow of current into the wire. This shows that in the system the charged ions would seek the easiest possible way to get grounded and when no other option is available the RWI starts to form.

In figure 6, we notice the increasing voltage with respect to increasing thickness which tells us that a higher ion energy is needed to start the instability, because the ions are farther from the plate electrode which results in a weaker force. So higher voltages, causing higher energy ions, are needed to create a significant force to start the instability. We also see in the thermal gradient that the higher energy ions bombard the central region of the instability and over there we also see a smaller cell size. In figure 12, we can view the corona streamer and the resulting gas flow. Using the schlieren method, we can also observe an increased temperature of gas flowing towards our oil due to residual gas heating and exothermic chemical reactions taking place due to ionization. This can explain the faster rate of temperature increase at the centre of the instability as seen between figure 10*b* and *c*, where the central temperature increases by 0.5°C while border temperature remains rather unchanged.

## 5. Conclusion

Instability of the air–liquid interface under an applied electric field has shown that the ions in effect of the electric field go towards the plane electrode. Unlike an air capacitor, in our point–plane configuration ions are produced which are charged. These ions flow towards the plate to be grounded but the dielectric layer in effect prevents them from doing so. Before the instability can occur, the charges accumulate on the surface of the oil and in effect of their relative energies, produce a certain force on the oil which when strong enough pushes the oil out of the way, This initial opening or weak spot in the oil is our initial dimple and this opening weakens further, increasing in size. The process of self-organization occurs in the oil to make the openings in essence the boundaries of polygons, which ensures stability of the system. As we see in figure 5, just as the dimple forms at the critical voltage, a current is detected in our system and that current increases as we increase voltage. If we recall from figures 7 and 8 we know that the cell size gets smaller and smaller with increased voltage which means more surface area for the ions to ground, and that supports our conclusion of the mechanism of the RWI. Furthermore, we also conclude that the ions are the main cause of the instability and image the system in two new perspectives (thermal and schlieren) which reveal previously unknown phenomena associated with the RWI.

## Data accessibility

Data are available at Dryad (http://dx.doi.org/10.5061/dryad.q680n) [8].
